# Integration of Industrially-Oriented Human-Robot Speech Communication and Vision-Based Object Recognition

**DOI:** 10.3390/s20247287

**Published:** 2020-12-18

**Authors:** Adam Rogowski, Krzysztof Bieliszczuk, Jerzy Rapcewicz

**Affiliations:** Department of Automation and Metal Cutting, Warsaw University of Technology, 02-524 Warsaw, Poland; krzysztof.bieliszczuk@pw.edu.pl (K.B.); jerzy.rapcewicz@pw.edu.pl (J.R.)

**Keywords:** human-robot speech communication, vision systems, industrial robotics

## Abstract

This paper presents a novel method for integration of industrially-oriented human-robot speech communication and vision-based object recognition. Such integration is necessary to provide context for task-oriented voice commands. Context-based speech communication is easier, the commands are shorter, hence their recognition rate is higher. In recent years, significant research was devoted to integration of speech and gesture recognition. However, little attention was paid to vision-based identification of objects in industrial environment (like workpieces or tools) represented by general terms used in voice commands. There are no reports on any methods facilitating the abovementioned integration. Image and speech recognition systems usually operate on different data structures, describing reality on different levels of abstraction, hence development of context-based voice control systems is a laborious and time-consuming task. The aim of our research was to solve this problem. The core of our method is extension of Voice Command Description (VCD) format describing syntax and semantics of task-oriented commands, as well as its integration with Flexible Editable Contour Templates (FECT) used for classification of contours derived from image recognition systems. To the best of our knowledge, it is the first solution that facilitates development of customized vision-based voice control applications for industrial robots.

## 1. Introduction

Human–machine voice communication is becoming an increasingly popular research topic. It is an important element of the Industry 4.0 concept which puts emphasis on efficient cooperation between man and machine [1]. Many aspects of direct human–robot cooperation are the subject of research and development, e.g., implementation of collaborative robots in automotive industry [2], robots learning skills from human demonstrations [3], security and human trust in security systems [4], social acceptance of robots working side-by-side with humans [5]. There are even attempts to adapt the techniques typically used with collaborative robots (like hand guiding) to traditional industrial robots [6].

Efficient collaboration needs efficient communication. The most natural means of communication between humans is speech. Therefore, it is desirable to establish a natural bidirectional voice communication between machines and their operators [7]. There have been many scientific experiments and even commercial implementations of such communication in areas like medical assistance, robotics, law enforcement, defense, aviation, home automation, security access control, etc. [8]. As far as robotics is concerned, reported solutions belong to categories like human–robot social interaction [9], teleoperation/telerobotics [10], surgical robots [11] or industrial robotics [12,13,14,15,16]. Speech recognition alone [10] or combined with gesture recognition [17] can be used to control mobile robots. Similar research was reported in respect to mobile cranes [18]. Erol et al. presented a robot voice control system based on Amazon’s Alexa Dot [19].

The effectiveness of voice communication between humans often depends on the context in which a verbal statement is uttered. Context in a general sense is any information used to characterize the situation of the subject. It can be derived from what has been shown or said earlier [20] but also from data provided by other senses (vision, touch). As far as human-robot voice communication is concerned, there is a need to develop methodologies that could help users to control and program a robot, with a higher level of abstraction from robot language [21]. This usually requires context to be involved. However, the art of context data depends on application. Some voice control systems do not need a context at all, because voice commands are simple (e.g., “left”, “right” etc.). This is the case of prosthetic robot arm presented by Gundogdu et al. [22]. A more advanced concept related to medical robots was described by Zinchenko et al. [11]. They propose an interesting solution: the way a voice command is uttered (duration of phonemes) influences the duration of a robot’s motion. As far as industrial applications are concerned, Li et al. developed a voice control system for a robotized flexible manufacturing cell, where simple, unambiguous commands like “Lathe, clamp the chuck” were used [23]. However, when robots have to act more autonomously and/or they operate in a complex environment, integration of speech recognition and other “artificial senses” is needed. Gustavsson et al. present results of their research on integration of speech communication and haptic control for collaborative robot [24]. In order to increase effectiveness of speech-based human-robot teamwork, Huang and Mutlu developed anticipatory robot control system based on user’s gaze monitoring [25]. Kharlamov and Ermishin [26] who deal with a mobile service robot, use sonars and laser scanner as a source of information on robot’s environment.

The most effective source of context data for voice commands are image recognition systems. Even simple social robots use both vision and speech recognition [27]. In recent years, significant research was devoted to integration of speech and gestures [17,28,29]. Maurtua et al. used a fusion of several interaction mechanisms to improve the performance of human–robot communication [30]. Like in our research, their aim was to allow system integrators and end-users to develop custom robotic systems that answer their needs. They created a modular industrial robotic solution, consisting of four modules (knowledge manager, voice interpreter, gesture interpreter, and fusion engine). Another interesting work dealing with speech and gesture integration was described by Yongda et al. [31]. Their method consists of analysis of voice commands to determine whether they contain enough information (orientation, distance) for the robot to perform its tasks. When information is insufficient, lacking data is derived from gesture recognition module.

Little attention, however, has been paid to vision-based identification of objects manipulated by industrial robots (like workpieces or tools) referred to by general terms used in voice commands. Hoppenstedt et al. dealt to some extent with integration of speech and image recognition, but their aim was different: application of voice commands to labeling input images used for training of image classifier [32]. Generally, there are no reports on any methods facilitating the abovementioned integration, although there are some examples of industrially oriented speech–vision fusion. For example, Tasevski et al. presented a complete solution for voice and vision integration (industrial robot picking and placing simple elements) [12]. However, their solution is not a general one because they restrict object recognition to few pre-defined shapes (basic geometrical figures). Similarly, elements of speech-vision fusion are present in the work by Bingol and Aydogmus [33], who developed a system for task-oriented speech-based control of an industrial robot by operators without any prior knowledge or experience in robotics. In their paper, they focus on voice control in Turkish language. Image recognition is restricted to red points or circles, marked on A4 paper, and symbolizing target points in machining process, e.g., drilling. Shaikh et al. [34] designed a simple voice-controlled prototype of automated personal assistant for domestic and industrial purposes. Image processing is incorporated in the form of object tracking (blue circle must be affixed to the target object). No general solution facilitating effective speech–vision fusion is presented.

Development of context-based voice control systems is a big challenge because image and speech recognition use different data structures, describing reality on different levels of abstraction. As pointed out by Kharlamov and Ermishin [26], the objects which interact with robots are usually described by coordinates while human commands are task-oriented. Besides, there are no universal algorithms for object classification, because they are application-specific. Depending on the application, two similar images may be interpreted as quite different objects. On the other hand, some objects of different geometric properties may belong to the same class. The best solution to this problem seems to consist in formulating the object classification rules by humans individually for each application. Unfortunately, this is a very labor-intensive and time-consuming task, unless it were a very simple case like that described in [35], where the presence of an object at a given position is a sufficient indication that it belongs to a given class. Therefore, the aim of our research was to develop a universal tool, facilitating formulation of classification rules for objects corresponding to general terms used in voice commands. Thus, development of customized context-based voice control applications will be much more easy and effective.

It is worth mentioning that the problem of context recognition for voice-controlled robots seems to be somehow related to issues encountered in CAD/CAM and CAPP systems. Automatic Feature Recognition (AFR) is also struggling with description incompatibility between systems. Losing information on object features is often a problem when converting 3D CAD models to a STEP file [36]. A very interesting study from our point of view was presented by Marchetta and Forradellas [37]. They developed a method that focused on the simplicity and expressiveness of feature representation in order to make customization of feature libraries easier and less costly. Although their research is related to different area and—of course—their results cannot be used for integration of image and speech recognition systems, yet the general idea was somehow similar.

Summarizing the state-of-the-art, it must be remarked that following issues have not been solved yet or only little attention have been paid to them:

- Although some works are reported that deal with fusion of speech and vision (mainly for specific applications), no general tool facilitating integration of industrially-oriented human-robot speech communication and vision-based object recognition has been developed so far. As development of context-based voice control systems is a laborious and time-consuming task, there is a need to create such a tool in order to allow system integrators and end-users to develop custom robotic systems that answer their needs.
- While significant research was devoted to integration of speech and gestures, little attention has been paid so far to vision-based identification of objects referred to by general terms used in voice commands.

The main innovation of our research consists in addressing these important issues. The remainder of this article is organized as follows: Section 2 presents general conception of industrially-oriented, context-based voice control system. The core of our method consists in integration of extended Voice Command Description Format (describing syntax and semantics of task-oriented commands) and Flexible Editable Contour Templates (used for classification of contours derived from image recognition system). This integration was achieved through development of appropriate form and structure of object identification rules. This issue is described in detail in Section 3. The algorithm for object identification and determination of its parameters is subject of Section 4. Section 5 presents laboratory implementation and discusses our experimental results. Finally, Section 6 contains our conclusions and plans for the future.

## 2. General Conception of Industrially-Oriented Context-Based Voice Control System

Unless the sublanguage used by a robot operator is a small set of strictly determined commands, development of industrially-oriented voice control systems may be a very challenging task for two reasons. Firstly, there is a need to take into consideration diversity of command formulations characteristic for natural way of speaking. Secondly, such sublanguage is usually application-oriented, hence it must be developed “from the scratch”. Therefore, there was a need to create a tool that would provide an easy way to define sublanguages used in individual applications. Its core was VCD format for description of syntax and semantics of voice commands [38]. A little part of sample sublanguage description below explains the essence of VCD:

**Code 1.** Sample code in VCD format. 

*#com move right*

move gripper *distance *units to the right: p3*p4

*#def distance*

*ten: 10*

*fifteen: 15*

*twenty: 20*

*#def units*

*millimeters: 1*

*centimeters: 10*

*decimeters: 100*



The description of sublanguage syntax and semantics in VCD is intuitive and not time-consuming. The content of single command (in this case “move right”) is a chain of words and phrases. Component phrases (“distance”, “units”) are described in separate statements (#def). Semantic information contained in each phrase is presented as a numerical value (or arithmetic expression) following the colon. Semantic analysis of command as a whole is possible due to formulas (in this case p3*p4) containing the variables p1,p2,p3,p4... referring to semantic data of component phrases. More details regarding VCD can be found in [38].

**Figure 1:**
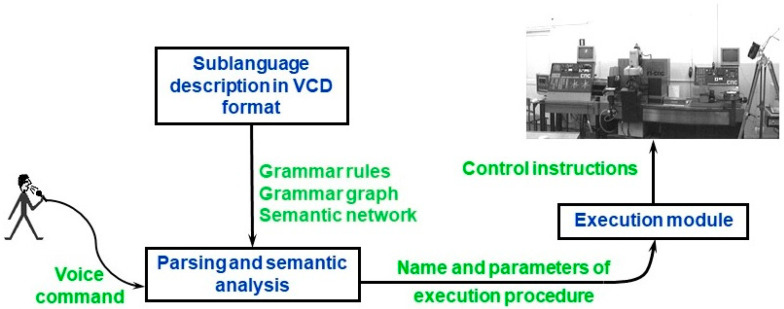
General scheme of command processing in contextless voice control system.

Voice command processing in contextless voice control system is performed according to the scheme presented in Figure 1. Based on the sublanguage description in VCD, appropriate data structures are automatically generated: the grammar rules required by speech recognition engine, grammar graph used by syntactic parser, procedural semantic network used in semantic analysis. As a result of command analysis, name and parameters of execution procedure are sent to the execution module. Detailed information can be found in [39].

For example, processing a command like:

“Move gripper twenty centimeters to the right”

Results in generating an execution procedure like:

“RIGHT (200)”

where *RIGHT* is the name of execution procedure, and 200 is its numerical parameter determining motion length in basic units (in this case millimeters), calculated as multiplication of values represented by 3rd (p3—“distance”) and 4th (p4—“units”) phrase of the command.

**Figure 2:**
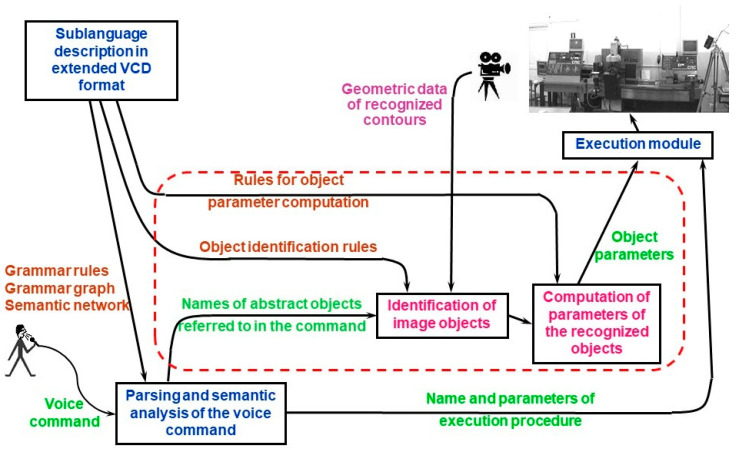
Integration of voice and image recognition systems.

Unfortunately, neither the VCD format nor the algorithms for voice command processing allow one to automatically incorporate the context data. The robot operator cannot use a command like “Robot, reach me the nut” because the term “nut” refers to an abstract object and—due to the lack of concrete data—it cannot be transformed into the set of numerical parameters needed by the execution module. In order to overcome this problem, appropriate data structures and algorithms would have to be individually developed for each voice control application. This is a very laborious task. Therefore, as a result of our current research, we have extended the VCD format and modified the algorithm for voice command processing in order to introduce so called object identification rules into the sublanguage description used in VCD. The extended VCD format makes it possible to define commands referring to abstract objects because identification rules for those objects are the part of the sublanguage description. Those rules let one automatically map the general terms used in voice commands into geometric objects derived from image recognition system. In this way, the objects referenced in commands are instantiated and they are assigned a set of numerical parameters determining their location, dimensions etc. When the user utters a command like “Reach me the nut”, automatic identification of proper object and calculation of desired gripper position is performed. General scheme of context-based robot voice control system is presented in Figure 2. The dashed line in Figure 2 surrounds elements of voice control system that are new against the previous conceptions.

As can be seen, in this new scheme the input to the execution module contains not only the name of execution procedure and its parameters resulting directly from semantic analysis, but also geometrical data of recognized object(s). Hence the execution procedure may refer to this data when generating control instructions for robot.

In order to incorporate the new elements (presented in Figure 2) into VCD-based sublanguage description, a modification was introduced into the structure of voice command heading against its old version described in [38]. The new VCD structure (defined in extended Backus-Naur form) is the following:

**Code 2.** Sublanguage description in extended VCD. 

*sublanguage description = set of statements;*

*set of statements = statement | set of statements, statement;*

*statement = command definition | phrase definition | escape expression | library reference;*

*command definition = command heading, set of rules;*

*phrase definition = phrase heading, set of rules;*

*command heading = ‘#com’,[space],command id,[‘/’,object list],new line;*

*object list = object reference|object list,’ ,’, object reference;*

*object reference = name;*

*command id = name;*

*phrase heading = ‘#def”, [space], phrase id, new line;*

*phrase id = name;*

*set of rules = rule | set of rules, rule;*

*rule = phrase sequence, [semantic data] , new line;*

*semantic data = ‘:’, set of expressions;*

*set of expressions = expression | set of expressions, delimiter, expression;*

*delimiter = ‘;’ | new line, ’;’ | ’;’ ,new line;*

*expression = ? arithmetic expression representing constant value ? | ? arithmetic expression containing variables p1,p2,p3… representing parameters returned by phrases 1,2,3… ?*

*phrase sequence = phrase | phrase sequence, space, phrase;*

*phrase = word sequence | phrase reference | optional phrase reference | wildcard sequence;*

*word sequence = word | word sequence, space, word;*

*word = obligatory word | optional word;*

*obligatory word = alphabetic string;*

*optional word = ‘?’, alphabetic string;*

phrase reference = ‘*’, phrase id, [ ‘(‘ , library name, ‘)’];

*optional phrase reference = ‘?’, phrase reference;*

*escape expression =’#esc’, [space], word sequence;*

*library reference = ‘#use’, [space], library file;*

*name = letter | name, letter | name, digit | name, ‘_’;*

*alphabetic string = letter | alphabetic string, letter;*

*library file = ? filename ?;*

*space = ? space character ?;*

*new line = ? new line character ?;*

*wildcard sequence = ‘…’;*

*digit = ‘0′ | ‘1′ | ‘2′ | ‘3′ | ‘4′ | ‘5′ | ‘6′ | ‘7′ | ‘8′ | ‘9′;*

*letter = ? alphabetic character ?;*



For example, a voice command referring to grasping one of nuts lying on the workbench, could be now defined in VCD as follows:

**Code 3.** Example of VCD-based voice command description.

*#com grasp/nut*

grasp the *which nut: p3

*#def which*

*left: 1*

*right: 2*



A new element, not available in the old (contextless) version of VCD (and now added as optional part of command heading) is a reference to object identification rules (which will be discussed in the next section). This reference has the form of *#obj* statement name (in this case: *nut*) preceded by forward slash. The mentioned *#obj* statement is heading of VCD segment containing the object description. In this way the object identification rules become a part of sublanguage description in VCD.

The terms used for description of various objects in voice commands are usually application-specific. So are the identification rules. This means that identification rules must be individually created for each application. Therefore any description of the rules must be transparent and easy to create. As it will be presented, the extended VCD format fulfills this condition.

## 3. Integration of Voice Command Description Format (VCD) and Flexible Editable Contour Templates (FECT)

As mentioned previously, the main aim of our research was to create a universal tool facilitating integration of industrially-oriented human–robot speech communication and vision–based object recognition in order to allow system integrators and end–users to develop custom robotic systems that answer their needs. Fusion of speech and vision according to the scheme shown in Figure 2 consists in integration of two main elements. The first one is VCD-based description of voice command sublanguage. The second one is set of object identification rules which allow to map the terms used in voice command into objects derived from image recognition system. As explained in Section 2, due to our extension of the VCD format, both elements may be created using common platform. Object identification rules use common syntax with VCD-based voice command description.

As far as object identification rules are concerned, they base on geometrical and topological relations between contours recognized by vision system. This is briefly discussed in Section 3.1, whereas details can be found in Section 3.3. Object identification rules refer to individual contour templates contained in a shape library. As the shape library is based on Flexible Editable Contour Templates (FECT) that were developed as a result of our previous research [40], we first included a short summary of FECT-based method in Section 3.2. in order to provide readers with the needed background. The object identification algorithm and object data structures are presented in detail in Section 4.

### 3.1. Conception of Object Identification Rules

As mentioned in Section 2, the heading of voice command description in extended VCD format may include a reference to object description which contains a set of identification rules. As depicted in Figure 2, identification rules refer to geometric data of object contours derived from a vision system. We make an assumption that each object in the image is represented by a set of elementary closed contours. Even if only incomplete edges are available (e.g., due to partial occlusions), Hough transform or other techniques may be applied in order to cope with this problem (this technical issue, however, does not belong to the scope of the current paper).

**Figure 3:**
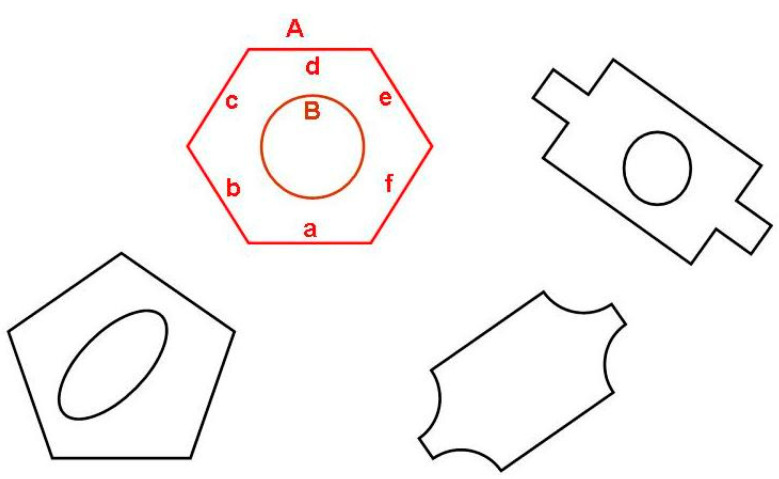
Sample image after contour retrieval. Contours A and B represent an object described by general term “nut”.

As pointed out by Yang et al. [41], topology and spatial relations among objects contained in images are crucial for image understanding. Therefore, object classification in our system is based on identification of individual contours representing the object (like e.g., contours A and B representing the object “nut” in Figure 3) as well as topological and geometrical relations between those contours and/or their segments (like a,b,c,d,e,f in the same figure).

Identification of individual contours is based on matching them against Flexible Editable Contour Templates (FECT) contained in shape library (see next subsection). As far as geometrical and topological relations are concerned, they have a form of sets of conditions that must be fulfilled. For example, contours A and B (understood as sets of points) may represent a nut when following conditions are fulfilled:

(1) $A\sim H_{T}$
(2) $B\sim C_{T}$
(3) A∩B={∅}
(4) $\forall P_{I}\in I\backslash B\;\exists P_{B}\in B\;:\;\left|\vec{P_{B}P_{I}}\cap A\right|\neq 0$
(5) $\forall P_{C}\in C\backslash \left(A\cup B\right)\;\exists P_{I}\in I\;:\;\vec{P_{C}P_{I}}\cap A=\left\{\emptyset \right\}$
(6) $\frac{\sum_{i=1}^{\left|A\right|}x\left(P_{A_{i}}\right)}{\left|A\right|}=\frac{\sum_{j=1}^{\left|B\right|}x\left(P_{B_{j}}\right)}{\left|B\right|}$
(7) $\frac{\sum_{i=1}^{\left|A\right|}y\left(P_{A_{i}}\right)}{\left|A\right|}=\frac{\sum_{j=1}^{\left|B\right|}y\left(P_{B_{j}}\right)}{\left|B\right|}$

where:

$I=\left\{P_{I}:P_{I}\;is\;any\;point\;on\;the\;image\right\}$
$C=\left\{P_{C}:P_{C}\;is\;point\;belonging\;to\;any\;contour\;on\;the\;image\right\}$
$H_{T}=\left\{P_{HT}:P_{HT}\;is\;point\;belonging\;to\;template\;of\;regular\;hexagon\;in\;the\;shape\;library\right\}$
$C_{T}=\left\{P_{CT}:P_{CT}\;is\;point\;belonging\;to\;template\;of\;circle\;in\;the\;shape\;library\right\}$
$P_{A{\;}_{i}}\in A$
$P_{B_{j}}\in B$

Providing an appropriate form in which such conditions could be described by the end-user in an easy and transparent way, as well as to maintain consistency with VCD format was a substantial challenge. An appropriate structure for object identification rules had to be developed. This is the topic of Section 3.3. However, we must explain the essence of the shape library first.

### 3.2. Shape Library

The object identification rules refer to individual contours constituting the images of objects to be manipulated by a robot. Those contours must be first correctly recognized by matching them against appropriate templates. Therefore, a library of contour templates (a shape library) must be available. As those templates are Flexible Editable Contour Templates (FECT) [40], the library can be easily created/modified by end-users. The essence of FECT consists of a flexible manner, in which contours consisting of elementary segments (straight lines, arcs, Bezier curves) are described. This flexibility results from two features of that description. Firstly, dimensions can be determined either unambiguously or as the ranges of allowable values. Secondly, relationships between dimensions can be determined using variables and/or arithmetic expressions. This lets one classify objects of even significant shape differences in one group. Still, there is no ambiguity of any kind in object classification because the rules are determined by user for each individual application. When a small difference in shape is crucial for distinction between objects belonging to different classes, this can be also included in the FECT.

FECT is described as a sequence of statements similar to “drawing instructions”, e.g., “Draw the straight line segment p of the length 50 to 100, continue with the arc q which is bent to the right. The angle of this arc is from 45 to 90 degrees…” etc. Details can be found in [40]. A simple example of FECT (template for regular hexagon shown in Figure 3) is presented below:

**Code 4.** An example of flexible template for regular hexagon. 

*#cnt regular hexagon*

*line: a/length: l*

*go: right/angle: 60*

*line: b/length: l*

*go: right/angle: 60*

*line: c/length: l*

*go: right/angle: 60*

*line: d/length: l*

*go: right/angle: 60*

*line: e/length: l*

*go: right/angle: 60*

*line: f/length: l*



The contour segments are assigned names (a, b, c, d, e, f in the above example) which may be later used by object identification rules. We note that image contours which match the above template may be regular hexagons of any dimensions, because hexagon side length is determined not by a concrete value but by a variable l. It means that while all hexagon sides must be of the same length, the length itself is not unambiguously determined.

In order to compare the contours on the image with Flexible Editable Contour Templates, various methods can be used. The most natural one consists in matching all individual contour segments. This requires the segments of contours in the image to be isolated first. Similarly like in the method presented by Seng [42], this contour recognition algorithm requires feature points (contour turning points) to be found. Contour matching itself is a two-stage process [40]. Rough matching takes into account only the structure of feature points and general direction of contour segments. Detailed matching involves comparison of signatures describing contours on the image and those in the template. Difference of signatures is equivalent to distance between points belonging to contour on the image and corresponding points in the template.

There is also the possibility to apply another method for contour comparison, based on an artificial neural network that lets one classify contours without reference to feature points [43]. This issue, however, does not fall within the scope of the current article.

### 3.3. Object Identification Rules

Creation of the object identification rules structure was the core of our current research aiming at development of the method for integration of industrially–oriented human-robot speech communication and vision–based object recognition. After the contours on the image provided by a camera have been recognized by matching them against templates in the shape library, object identification rules can be applied in order to recognize (classify) the objects represented by those contours. The structure of object identification rules is presented below in extended Backus-Naur form:

**Code 5.** Structure of object identification rules. 

*identification rules library = description of objects;*

*description of objects = object description|description of objects,object description;*

*object description = heading,description of contours[,description of parameters];*

*heading = ‘#obj’[,space],object name,new line;*

*object name = name;*

*description of contours = component contours[,exclusions];*

*component contours = declaration of contours[,identification rules];*

*declaration of contours = ‘contours’,list of contours,new line;*

*exclusions = excluded contours|exclusions,excluded contours;*

*excluded contours = declaration of excluded contours[,identification rules];*

*declaration of excluded contours =‘ exclude’,list of contours,new line;*

*list of contours = contour declaration|list of contours,‘,’,contour declaration;*

*contour declaration = contour name,‘(‘,contour id,‘)’|contour name,‘,’,contour declaration;*

*contour name = name;*

*contour id = ?contour id in the shape library?|‘any’;*

*identification rules = identification rule|identification rules,identification rule;*

*identification rule = ?topological or logical condition?,new line | auxiliary definition,new line;*

*auxiliary definition = ’aux’,name,’=’,?function returning point or line?;*

*description of parameters = declaration of parameters,calculation rules;*

*declaration of parameters = ‘parameters’,list of parameters,new line;*

*list of parameters = parameter declaration|list of parameters,‘,’,parameter declaration;*

*parameter declaration = parameter name,‘(’,parameter type,‘)’|parameter name,‘,’,parameter declaration;*

*parameter name = name;*

*parameter type = ‘real’|‘point’|‘position’;*

*calculation rules = assignment|calculation rules,assignment;*

*assignment = ?parameter identification?,‘=’,?arithmetic expression?,new line;*

*name = ?string consisting of alphabetic characters and digits?;*

*new line = ?new line character?;*

*space = ?space character?;*



As can be seen, description of an individual object may generally consist of following parts:

- heading
- list of contours composing the object,
- conditions involving contours composing the object
- list of “excluded” contours,
- conditions involving “excluded” contours,
- list of object parameters,
- rules for calculation of object parameters.

Contours declared with the identifier ‘any’ may be of any type. “Excluded” contours are the contours that must not be components of a given object. Introduction of such elements makes the object identification rules more flexible. A sample description of an object referred to by general term *nut* (see Figure 3) is presented below:

**Code 6.** A nut description in extended VCD format. 

*#obj nut*

*contours A (hexagon), B (circle)*

*inside (B, A)*

*equal (A .centroid, B .centroid)*

*exclude C (any)*

*inside (C, A)*

*parameters D (real)* *, grasp (position)*

*D = radius (B)*

*grasp .point = B .centroid*

*grasp .angle = A .a.angle*



The first line is the object description heading (#obj nut). The second line determines that two contours (hexagon *A* and circle *B*) that compose the object image. This line refers to FECTs: *hexagon* and *circle* in the shape library. Lines 2 through 5 contain conditions involving contours *A* and *B*. Line 3 determines that contour *B* must be inside of contour *A*. Expression in line 4 means that centroid of both contours should coincide (within some tolerance). Lines 5 and 6 contain a condition that no other image contours are allowed to be inside the hexagon A.

Line 7 contains the declaration of two “object parameters” (radius D of the hole *B* and gripper position *grasp* appropriate for grasping the nut). We must bear in mind that identification of objects is not the final goal of any robot control system. The final goal is always determination of robot gripper coordinates, corresponding to appropriate grasp points. There are even examples of research aimed at grasp point determination without prior object segmentation [44]. Therefore, the object identification rules in extended VCD format contain not only conditions allowing to identify the objects to be manipulated by robot, but also the rules for calculation of “object parameters” (in the above example those rules are contained in lines 8 through 10). “Object parameters” are variables that provide information about the object, meaningful from the point of view of execution module e.g., desirable position of the gripper (like variable *grasp* in the above example).

Generally, identification rules may be expressed in the form of conditions involving contours as a whole (e.g., *inside (A, B)*), contour segments (e.g., parallel (A.a, A.d)) as well as their parameters e.g., equal (A .centroid.x, B .centroid.x). Logical operators like *AND*, *OR*, *XOR* can be employed. The rules may use arithmetic expressions containing basic operators (e.g., +, −, *,/) and functions (e.g., *distance (A.a, A.d)*). Due to the lack of space it is impossible to present them all here. Therefore, we explain here only selected functions, representing individual function types (more information can be found in Supplementary Materials Research data S1):

- Real function *distance(A, B)* calculates distance between two geometrical objects (contours, segments, points) according to the expression: $distance\;\left(A,B\right)\;=\;min\left|P_{A}-P_{B}\right|\;where\;P_{A}\in A\;and\;P_{B}\in B$.
- Logical function *inside(A, B)* checks whether the object A is inside the object B: $inside\left(A,B\right)\;⟺\forall P_{I}\in I\backslash A\;\forall P_{A}\in A\;:\;\left|\vec{P_{A}P_{I}}\cap B\right|\neq 0\;where\;I=\left\{\;P_{I}:P_{I}\;is\;any\;point\;on\;the\;image\right\}$.
- Function ***centroid(A)*** returns a point being centroid of geometrical object A: $centroid\left(A\right)=\left(\frac{\sum_{i=1}^{\left|A\right|}x\left(P_{A_{i}}\right)}{\left|A\right|},\frac{\sum_{i=1}^{\left|A\right|}y\left(P_{A_{i}}\right)}{\left|A\right|}\right)where\;P_{A_{i}}\in A$.

Sample identification rules for various objects can be found in Supplementary Materials Research data S2.

## 4. Object Identification Algorithm

**Figure 4:**
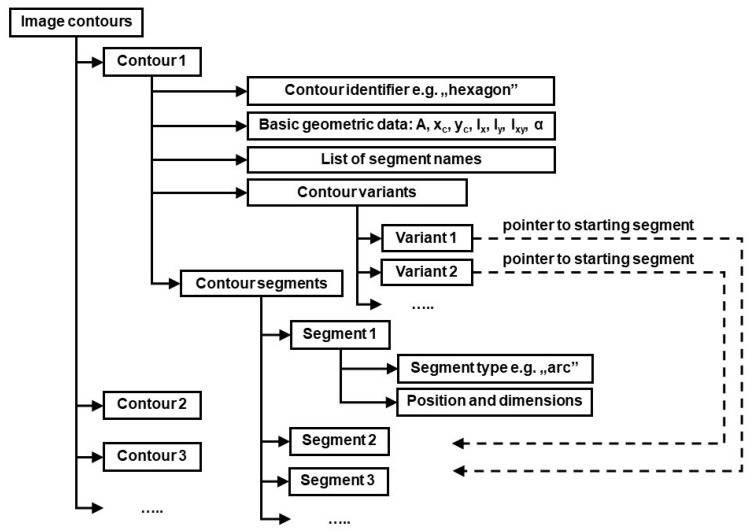
Data structure of identified image contours.

In order to identify objects referred to by general terms used in voice commands, all individual contours on the image provided by camera must be analyzed first. The basic geometric data of those contours is computed (area, centroid coordinates, second order central moments i.e., moments of inertia and product of inertia, the angle of principal axis α). This data may be later needed for calculation of output values of previously mentioned functions included in arithmetic expressions used in identification rules. Next, the contours on the image are identified by matching them against FECT templates in the shape library according to the algorithm presented in [40]. The structure of generated data is shown in Figure 4.

There is a need for an explanation regarding “contour variants” in the structure describing individual contours in Figure 4. Namely, as a result of template matching, each contour is assigned a list of segment names, according to description in FECT. Generally, the first name should correspond to “Segment 1”, the second name to “Segment 2” and so on. However, the very nature of Flexible Editable Contour Templates lets assign contours of different dimensions and shape proportions to the same class. For example, a rectangular contour will always match appropriate template, independent of which side (the longer or the shorter one) is considered the first contour segment. Sometimes individual contour segments cannot be matched unambiguously against corresponding template segments also because of contour’s shape regularity. An example is the regular hexagon shown in Figure 3. Each of six contour segments can be considered the starting one (i.e., named “a”). However, this ambiguity could later lead to false results regarding fulfillment of conditions imposed by object identification rules (the rules will often refer to segment names e.g., the rule may require the segment “a” of contour *A* to be parallel to segment *d* of contour *D*: parallel (A.a, B.d)). To cope with this problem, the data structure shown in Figure 4 includes contour variants. Each contour variant is determined by its starting segment.

**Figure 5:**
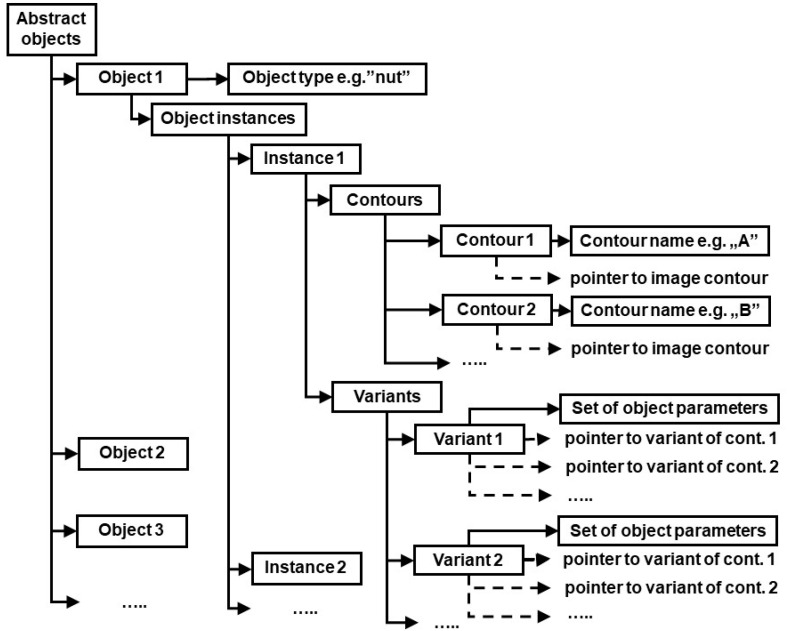
Data structure of recognized objects.

In the next step, the data structure shown in Figure 5 is generated using object identification rules. For each abstract object, its potential instances are determined through the search of the structure shown in Figure 4. If all conditions contained in identification rules are fulfilled, the contours belonging to current instance are assigned pointers to appropriate variants of image contours. If those conditions are not fulfilled, the current instance is deleted. Finally, the rules for calculation of object parameters are applied, and results of those calculations are assigned to each variant of each instance of all objects (see “Set of object parameters” in Figure 5). Created data is then made available to execution module which automatically generates control instructions for the robot. Algorithm responsible for generating data shown in Figure 5 is presented below:

**Code 7.** Algorithm generating data of recognized objects. 

***Algorithm 1:*** *Application of object identification rules*

***Inputs*** *:*

*Set D = {d: d is data record of single contour in the image} % see Figure 4*

*Set A = {a: a is abstract object referred to in voice command}*

*Set R = {r: r is object identification rule}*

***Output:*** *Set O = {o: o is data record of abstract object} % see Figure 5*

*k ← |A|*

*for i = 1 .. k do*

*create new object record o_i_*

*type (o_i_) ← type (a_i_)*

*r_i_ ← find object description for type (a_i_) in the library of identification rules R*

*m ← based on description r_i_, determine the number of contours constituting object a_i_*

*match ← true % auxiliary logical variable for verification of valid identification*

*n ← 0 % number of instances of abstract object a_i_*

*for each combination of m contour records d Є D do*

*n ← n+1*

*create new instance inst_n_ of object a_i_*

*for j = 1..m do*

*create new record for object contour description ocd_j_ % see Figure 5*
 *name (ocdj) ← determine name for j-th contour according to ri* *pointer(ocdj) ← determine address of data record d Є D of j-th contour* *if contour id (pointer (ocdj)) ≠ type (name (ocdj)) then* *match ← false* *end if* *end for* *if match then* *v ← 0 % number of object variants for instance instn* *for all combinations of all variants of image contours (Figure 4) referred by instn do* *v ← v+1* *create new variant record varv for instn* *for j = 1..m do* *pointer j (varv) ← determine pointer to current variant of contour referred by* *pointer(ocdj)* *end for* *match ← check all conditions contained in ri in regard to instn for variant varv* *if not match then* *delete variant record varv* *v ← v-1* *end if* *end for* *if v = 0 then* *match ← false* *else* *match ← true* *end if* *end if* *if not match then* *delete instance instn* *n ← n-1* *end if* *end for* *for j = 1..n do* *for m = 12..v do* *set of object parameters (varm (instj(oi))) ← apply the rules for object parameter calculation* *end for* *end for*  *end for*


## 5. Experimental Results

**Figure 6:**
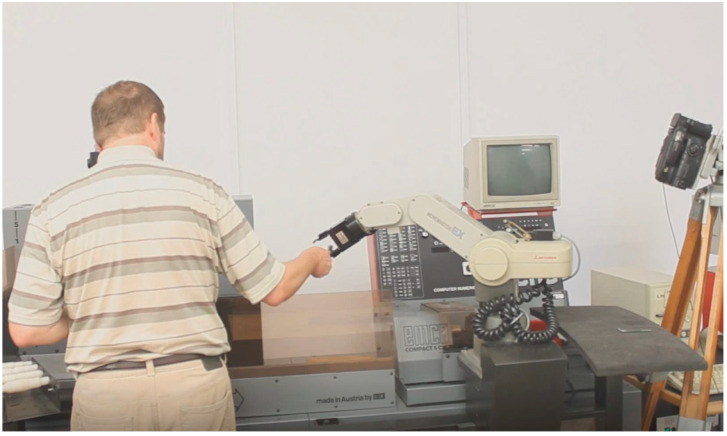
Robot-human collaboration in educational robotized cell.

The most important advantages of our method are mostly qualitative and thus not easy to measure. Our approach lets the end-users develop more robust customized voice control applications for industrial robots much easier and swiftly than before. The use of vision-based context recognition makes voice control system much more user-friendly and flexible. However, some aspects of the method can be also verified experimentally. In our laboratory, a voice control system was developed using educational robotized manufacturing cell. Its aim was to provide voice communication between operator and collaborative robot pursuing common goals. The robot’s task was to hand appropriate tools to the operator as well as to take away the tools which were no longer needed (Figure 6).

CCD cameras were connected to a custom image recognition system that fulfilled all requirements presented in this paper. The speech recognition system was based on Microsoft SAPI and employed VCD format for voice command language description. Of course, our method cannot be reduced to any individual implementation, but functioning of such implementation is a form of partial validation of the method as a whole.

The whole history of implementation of results of our past and current research regarding speech communication between humans and collaborative robots was following: Initially, a contextless system was developed. There were two possible solutions for such system to function properly. Either the positions and orientations of objects needed by operator had to be explicitly fixed (this was—of course—a source of substantial limitations) or the operator had to describe those positions orally (this resulted in complicated voice commands). Next, recognition of single contours using FECT was introduced in order to take into account the context provided by image recognition system. Finally, our current research made it possible to create the object identification rules and to incorporate them into voice command language description. In this way, the robot may perform orally uttered commands which use general terms referring to objects which are placed in any position.

In order to compare context-based and contextless speech communication, two variants of voice control system were used in our current experiment. In both cases robot’s tasks were the same, although the ways they were performed differed slightly. Common assumptions for both variants were as follows:

- The following tools were used: open end wrenches of various sizes, box end wrenches of various sizes, combination wrenches (one end was an open end and the other one was a box end), adjustable wrenches of two sizes (big and little), Allen (hex) keys of various sizes, special wrench (with more than two ends). In each experiment there were approximately 6-7 various tools lying simultaneously on the table.
- The task of the robot was to hand over the tools to the operator (on oral demand), as well as to take them away and place them again on the table. When the operator ceased to use a tool, he put it on the workbench for later use. If the tool was not expected to be needed in the nearest future, the operator could ask the robot to take the tool away from the workbench and put it on the table.
- The experiments were conducted under various circumstances: either without any noise or in the presence of noise caused by another man working nearby (e.g., hammering nails with different intensity levels).

In the contextless application it was not possible to automatically detect positions and orientations of the tools, hence they had to be placed in strictly pre-determined places on the table. Therefore, a sample command for handing over a tool by the robot could be like this: “Give me the hex wrench size eight” (tool type and size unambiguously determined the position). Even more complex commands were used for removing the tools from the workbench and putting them on the table. As the contextless system does not use image recognition, the tools had to be placed by the operator in strictly determined places on the workbench (those places were appropriately marked on the benchmark and assigned conventional numbers 1 to 4). In order to properly perform its task, the robot had to be informed both about the current position of the tool on the workbench as well as its type and size (these two values determined position where it should be placed on the table). It was namely important that the robot put back the tools exactly in original positions. Otherwise, it would be impossible for robot to grasp them when the operator needed them again. Therefore, a sample command could be formulated like this: “Take away the open-end wrench size fourteen from position three”. Unfortunately, such commands are long and therefore prone to be recognized incorrectly. Sublanguage description in VCD format for contextless version of our system is presented below.

**Code 8.** Syntax and semantics of sublanguage for contextless version. 

*#com give*

give me ?the *type_and_size: p4[1],p4[2]

give me ?the *big_little adjustable ?wrench: 5,p4

*give me ?the special ?wrench: 6,0*
 
*#com take*

take away ?the *type_and_size *from: p4[1],p4[2],p5

take away ?the *big_little adjustable ?wrench *from: 5,p4,p7

take away ?the special ?wrench *from: 6,0,p6
 
*#def type and size*

open end ?wrench ?size *size: 1,p5

box end ?wrench ?size *size: 2,p5

combination ?wrench ?size *size: 3,p4

*allen ?wrench ?size *sizeallen: 4,p4
 
*#def allen*

*allen*

*hex*
 
*#def size*

*ten: 10*

*twelve: 12*

*fourteen: 14*

*seventeen: 17*
 
*#def sizeallen*

*eight: 8*

*ten: 10*

*twelve: 12*
 
*#def big_little*

*big: 1*

*little: 2*
 
*#def from*

from position ?number *number: p4

in position ?number *number: p4
 
*#def number*

*one: 1*

*two: 2*

*three: 3*

*four: 4*



In the context-based version, speech communication was integrated with vision-based object recognition. Two cameras were installed: one above the table and one above the workbench. In this manner both the tools on the table as well as those put by the operator on the workbench were in the field of view. Neither of them had to be placed in strictly determined positions because all necessary data: the type of the tool, its size, as well as grasping position could be determined automatically according to the object identification rules. As far as voice command language is concerned, its description in VCD format is presented below.

**Code 9.** Syntax and semantics of sublanguage for context-based version. 

*#com give/open, box, combination, allen, adjustable, special*

give me ?the *type_and_size: p4[1],p4[2]

give me ?the *big_little adjustable ?wrench: 5,p4

*give me ?the special ?wrench: 6,0*
 
*#com take/open, box, combination, allen, adjustable, special*

take away ?the *type_and_size: p4[1],p4[2]

take away ?the *big_little adjustable ?wrench: 5,p4

*take away ?the special ?wrench: 6,0*

take away ?the *type ?wrench: p4,0

*take ?the wrench away: 0,0*

*take it away: 0,0*
 
*#def type_and_size*

open end ?wrench ?size *size: 1,p5

box end ?wrench ?size *size: 2,p5

combination ?wrench ?size *size: 3,p4

*allen ?wrench ?size *sizeallen: 4,p4
 
*#def type*

*open end: 1*

*box end: 2*

*combination: 3*

*allen: 4

*adjustable: 5*

*special: 6*
 
*#def allen*

*allen*

*hex*
 
*#def size*

*ten: 10*

*twelve: 12*

*fourteen: 14*

*seventeen: 17*
 
*#def sizeallen*

*eight: 8*

*ten: 10*

*twelve: 12*
 
*#def big_little*

*big: 1*

*little: 2*



As can be seen, both “give” and “take” commands refer to identification rules of objects called “open”, “box”, “combination”, “allen”, “adjustable”, “special”. As far as “give” command is concerned, its syntax is the same as in the contextless version because the same information is needed to unambiguously determine the tools needed by operator. Still, although the speech communication between operator and robot has the same form, the collaboration is much more comfortable because the tools can be placed in any position within camera’s field of view.

As far as “take” command is concerned, substantial advantages regarding command syntax are apparent. First of all, information about tool position on the workbench is no more needed because it can be derived from vision system according to object identification rules. Therefore, commands like “take away box end wrench size seventeen” are sufficient. Moreover, depending on the situation, we can use variant four (e.g., “take away the adjustable wrench”), when there is only one wrench of this type on the workbench, variant five (“take the wrench away”) or variant six (“take it away”) when there is only one tool on the workbench. In contextless version it was impossible. As can be seen, implementation of our method results in more concise i.e., shorter voice commands. Therefore, those commands can be recognized with greater reliability. We compared experimentally the recognition rates for both sets of above discussed voice commands. The results are presented in Table 1. Each variant included 50 spoken commands. All experiments except variant 1 were conducted in a noisy environment.

As it was not difficult to forecast, the recognition rate was better for context-based commands. However, experimental result show that this difference for contextless and context-based systems is significant. It is of particular importance in a noisy industrial environment. This shows that application of our method, which lets the end-users develop customized context-based voice control applications for industrial robots in an easy way, could be an important factor facilitating introduction of speech-based human-robot interfaces into industrial practice.

Of course, it is possible to simplify commands also in the contextless version. For example, when we assume that the operator always puts back the tools of each type and size in different, strictly determined positions, a little bit simpler commands like “take away the combination wrench of size fourteen” would be possible for contextless speech communication, too. However, it will be at the cost of convenient operation. The experiments show that—apart from recognition rate improvement —introduction of context-based speech communication results also in qualitative changes regarding effectiveness of human-robot collaboration. When the operator wants to put back the tool, he is no more required to wait for robot to grasp it, neither is he required to put back the tool in strictly determined position in order to let the robot lift it off. The only disadvantage of context-based systems in the past was laborious and time–consuming development of those systems. Our method lets minimize time and effort needed to develop a new application. For example, the voice control system used in the experiment described in this section needed several hours to be created, tested, and ready to use.

## 6. Discussion

The main contribution of research presented in this paper is a novel method for integration of human-robot speech communication and vision-based context recognition. The most important innovation aspects are the following:

- Our aim was to facilitate development of customized vision-based systems for voice control of industrial robots. Contrary to most other works, our solution does not consist only in a fusion of speech and vision for a particular application, but rather it provides users with a general tool for the integration of human-robot speech communication and vision-based object recognition.
- Contrary to other papers, we not only describe the method, but we also develop a ready-for-use metalanguage for description of voice command languages for context-based speech communication.
- Contrary to most of works which focus mainly on integration of speech and gestures, we fill the gap in research dealing with vision-based identification of objects referred to by general terms used in voice commands.

Due to the incorporation of object identification rules into our voice command language description, the development of customized, context-based voice control applications for industrial robots will be significantly easier and less time-consuming.

Whereas speech communication between humans and various electronic devices (computers, smartphones) is becoming more and more popular, industrial implementation of human-machine speech-based interaction still faces many obstacles. The following issues determine the human-machine communication prospects in industrial environment:

- Restricted reliability of speech recognition engines, particularly in the presence of noise. Industrial environment is generally very noisy and requirements regarding speech recognition rate are usually higher than in other areas because misunderstanding between human and machine may result in severe damage of equipment or even in a threat for human health and life.
- Speech communication effectiveness. Speech is the most natural means of communication for humans. However, this is true only under circumstance that natural language is used. When speech recognition system requires the user to utter unnatural, complicated sentences, the voice control will not be perceived as facilitation.
- Influence of human-machine speech communication on the whole human-machine collaboration process. For example, as shown in previous section, depending on the version of voice control sublanguage, the operator was either allowed to put the tools anytime and anywhere on the table or he was restricted both spatially and temporally. This may influence also effectiveness of the whole production process.

The use of context-based speech communication seems to improve the prospects in all these three areas. Our experiments showed that use of vision-based context might influence the speech recognition rate because voice commands are concise and simple. There is still a problem with the presence of noise, nevertheless the recognition rate is higher for context-based commands, hence such systems are more likely to be employed in industry when further improvements of speech recognition engines will be achieved.

However, improvement of speech recognition reliability is not the only advantage of context-based speech communication. The sublanguage consisting of more natural voice commands will probably make potential users perceive the human-robot voice communication as a useful tool. Additionally, as stated before, context-based voice communication will improve user’s comfortability and production process effectiveness. Therefore, we are convinced that our method may facilitate a broader introduction of man-machine speech interfaces into industrial practice in the very near future.

Besides, our solution is potentially much more general. It does not have to be restricted to communication between industrial collaborative robots and their operators. It may be also useful in the area of assistive robots. One of their tasks is handing over the objects that are beyond the grasp of disabled people. Those objects are often placed randomly, hence their positions should be determined using an image recognition system. As the types of the objects manipulated by assistive robots may vary, depending on individual situations, our solution may be very useful for customizing speech communication between disabled persons and assistive robots. Moreover, as sublanguage of voice commands uttered by disabled persons should be as natural as possible, the use of context-based speech recognition seems to be unavoidable.

However, our method in its current version still has some shortcomings. One of them results from the fact that its implementation is based on 2D image recognition. Although in many industrial applications this may be enough (e.g., when objects referred to in voice commands lie on a flat surface of known elevation), nevertheless there is sometimes a need to determine all coordinates (both linear as well as angular) describing the position of the object in question. Besides, sometimes correct recognition and classification of an object cannot be based on one viewpoint only. Therefore, our future plans involve extension of the method to 3D object identification with the help of multi-camera vision systems.

## Figures and Tables

**Table 1:** Recognition rates for voice commands.

Variant Number	Recognition Rate without Context [%]	Recognition Rate with Context [%]
1	100	100
2	72	100
3	86	98
4	60	94
5	66	86

## Supplementary Materials

The following are available online at https://www.mdpi.com/1424-8220/20/24/7287/s1, Research data S1: RULES.doc, Research data S2: SAMPLES.doc.
