# Supplementary material for: Integration of Industrially-Oriented Human-Robot Speech Communication and Vision-Based Object Recognition
Source: Sensors (Basel). 2020 Dec 18;20(24):7287. doi: 10.3390/s20247287 (PMC7767307; doi:10.3390/s20247287)
Supplement: Supplementary file 1 [file sensors-20-07287-s001.zip › RULES.doc]

**ELEMENTS OF SYNTAX OF OBJECT IDENTIFICATION RULES**

**Variable types:**

- REAL

**Application example:**

*parameter D (real)*

*D = 10*

- POINT

**Structure elements:**

x, y (real)

**Application example**:

*parameter P (point)*

*P.x = 10*

*P.y = 20*

- LINE

**Explanation:**

Line is an auxiliary entity, created with the use of AUX statement in order to facilitate definition of relationships between other objects. This entity itself will not be considered during object classification

**Application example**:

*Contours A,B (circle)*

*aux L = line (10,20,10,200)*

*symmetrical (A, B, L)*

- POSITION

**Structure elements:**

x, y, angle (real), point (point)

**Application examples**:

*parameter POS (position)*

*POS.x = 10 {or: POS.point.x = 10 }*

*POS.y = 20 {or: POS.point.y = 20}*

*POS.angle = 60*	{angle in degrees}

*POS.point = P*

**Image objects:**

- CONTOUR

**Structure elements:**

centroid (point), A, xc, yc, Ix, Iy, Ixy, alpha (real), segment names

**Explanation:**

A – area enclosed by contour

xc, yc – centroid (center of mass) coordinates

Ix, Iy, Ixy – second order moments of inertia

**Application example:**

*contour A (hexagon)*

*parameters X,Y (real)*

*X = A.centroid.x*

*Y = A.yc*

- SEGMENT

**Structure elements:**

startpoint (point), angle [, center, radius] (real)

**Application example:**

*contour B (external)*

*parameter P (point)*

*P = B.arc1.center*

**Functions returning real value:**

- DISTANCE (A, B***)***

**Parameters:**

A,B (contour | segment | line | point)

**Explanation:**

Calculates distance between two geometrical objects according to the expression: *distance(A,B)* = where and

- INCLINATION(A,B)

**Parameters:**

A,B (segment)

**Explanation:**

Checks whether segments A and B are lines and calculates angle between them:

where and are slopes (angles given in image global coordinate system) of lines A and B, respectively

- LENGTH(A)

**Parameters:**

A (segment)

**Functions returning logical value:**

(note that all functions listed below compares values with some engineering fit, by default 2%, this parameter is adjustable)

- INSIDE (A, B)

**Parameters:**

A (contour | segment | point), B (contour)

**Explanation:**

Checks whether the object A is inside of the object B:

where:

- BELONGS (A, B)

**Parameters:**

A (point), B (line | segment | contour)

**Explanation:**

Checks whether the point A belongs to object B

- PARALLEL (A, B)

**Parameters:**

A, B (segment)

**Explanation:**

Checks whether two segments are parallel by comparing their slope derived from image processing (the slope has to be the same)

- PERPENDICULAR (A,B**)**

**Parameters:**

A, B (segment)

**Explanation:**

Checks whether two segments are perpendicular by comparing their slope derived from image processing (the slope has to differ by 90 or 270 degrees).

- EQUAL (A, B)

**Parameters:**

A, B (real | point | position)

**Explanation:**

Checks whether two values are equal or whether two structures have equal parameters

- GREATER (A,B)

**Parameters:**

A, B (real)

**Explanation:**

Checks whether the first parameter is greater than the second one

- LESS (A,B)

**Parameters:**

A, B (real)

**Explanation:**

Checks whether the first parameter is less than the second one

- SYMMETRICAL (A,B,L)

**Parameters:**

A, B (point | line | segment | contour), L (line | segment)

**Explanation:**

Checks whether the objects A and B are symmetrical, where the line of symmetry is L

- BETWEEN (A,B1,B2)

**Parameters:**

A, B1, B2 (point | contour)

**Explanation:**

Checks whether the point A lies between two lines, perpendicular to line passing through points B1 and B2, where one of those lines is passing through point B1, and the other one passing through point B2. When A, B1, or B2 are contours, the above conditions refer to centroids of those contours

**Function returning points:**

- MIDPOINT (A)

**Parameters:**

A (segment)

**Explanation:**

Returns cross point of the segment and its bisector

- INTERSECTION (A, B)

**Parameters:**

A, B (line | segment | contour)

**Explanation:**

Returns intersection point of two geometrical entities

**Functions returning lines:**

- LINE (A, B)

**Parameters:**

A, B (point)

**Explanation:**

Returns auxiliary line passing through two points

- LINE (A, b)

**Parameters:**

A (point), b(real)

**Explanation:**

Returns auxiliary line passing through the point A and with the slope b (angle given in image global coordinate system)
