# Supplementary material for: Integration of Industrially-Oriented Human-Robot Speech Communication and Vision-Based Object Recognition
Source: Sensors (Basel). 2020 Dec 18;20(24):7287. doi: 10.3390/s20247287 (PMC7767307; doi:10.3390/s20247287)
Supplement: Supplementary file 1 [file sensors-20-07287-s001.zip › SAMPLES.doc]

**SHAPE LIBRARY (excerpt)**

#CNT RECTANGLE

LINE: a / LENGTH: L1

GO: LEFT /ANGLE: 90

LINE: b / LENGTH: L2

GO: LEFT /ANGLE: 90

LINE: c / LENGTH: L1

GO: LEFT /ANGLE: 90

LINE: d / LENGTH: L2

#CNT SQUARE

LINE: a / LENGTH: L

GO: LEFT /ANGLE: 90

LINE: b / LENGTH: L

GO: LEFT /ANGLE: 90

LINE: c / LENGTH: L

GO: LEFT /ANGLE: 90

LINE: d / LENGTH: L

#CNT CIRCLE

ARC: a / BEND: LEFT, ANGLE: 360

#CNT BLOCK

LINE: a / LENGTH: L1

ARC: f1 / BEND: LEFT, ANGLE: 90

LINE: b / LENGTH: L2

ARC: f2 / BEND: LEFT, ANGLE: 90

LINE: c / LENGTH: L1

ARC: f1 / BEND: LEFT, ANGLE: 90

LINE: d / LENGTH: L2

ARC: f1 / BEND: LEFT, ANGLE: 90

**BINARIZED IMAGES OF INDIVIDUAL OBJECTS (samples)**

PLATE:

BLOCK16

BLOCK22

BLOCK43

**VOICE COMMAND LANGUAGE DESCRIPTION IN VCD**

#COM GRASP PLATE / PLATE

GRASP *WHICH PLATE: p2

#DEF WHICH

BIG: 1

LITTLE: 2

MEDIUM: 3

#OBJ PLATE

CONTOURS E (RECTANGLE), Q1, Q2 (SQUARE), C1, C2, C3, C4 (CIRCLE)

GREATER ( LENGTH (E.a), LENGTH (E.b) )

AUX AXIS1 = LINE ( MIDPOINT (E.d), MIDPOINT (E.b) )

AUX AXIS2 = LINE ( MIDPOINT (E.a), MIDPOINT (E.c) )

INSIDE ( Q1, E )

INSIDE ( Q2, E )

INSIDE ( C1, E )

INSIDE ( C2, E )

INSIDE ( C3, E )

INSIDE ( C4, E )

PARALLEL ( Q1.a, AXIS1 )

PARALLEL ( Q2.a, AXIS1 )

SYMMETRICAL ( Q1, Q2, AXIS1 )

SYMMETRICAL ( C1, C2, AXIS1 )

SYMMETRICAL ( C3, C4, AXIS1 )

SYMMETRICAL ( C1, C3, AXIS1 )

SYMMETRICAL ( C2, C4, AXIS1 )

BELONGS ( Q1.CENTROID, AXIS2 )

PARAMETERS SIZE (REAL), GRASP (POSITION)

SIZE = DISTANCE ( E.b, E.d )

GRASP.POINT = E.CENTROID

GRASP.ANGLE = E.a.ANGLE

#COM GRASP DOMINO / BLOCK16, BLOCK22, BLOCK43

GRASP *NUMBER *NUMBER DOMINO BLOCK: p2, p3

#DEF NUMBER

ONE: 1

TWO: 2

THREE: 3

FOUR: 4

FIVE: 5

SIX: 6

#OBJ BLOCK16

CONTOURS E (BLOCK), M (RECTANGLE), L, R1, R2, R3, R4, R5, R6 (CIRCLE)

INSIDE ( L, E )

INSIDE ( M, E )

INSIDE ( R1, E )

INSIDE ( R2, E )

INSIDE ( R3, E )

INSIDE ( R4, E )

INSIDE ( R5, E )

INSIDE ( R6, E )

GREATER ( LENGTH (E.a), LENGTH (E.b) )

LESS ( LENGTH (M.a), LENGTH (M.b) )

PARALLEL ( M.a, E.a )

AUX AXIS1 = LINE ( MIDPOINT (E.d), MIDPOINT (E.b) )

AUX AXIS2 = LINE ( MIDPOINT (E.a), MIDPOINT (E.c) )

BELONGS ( MIDPOINT ( M.a), AXIS2 )

SYMMETRICAL ( R1, R2, AXIS1 )

SYMMETRICAL ( R3, R4, AXIS1 )

SYMMETRICAL ( R5, R6, AXIS1 )

BELONGS ( L.a.CENTER, AXIS1 )

BETWEEN ( L, MIDPOINT (E.d), M )

BETWEEN ( R1, M, MIDPOINT (E.b) )

BETWEEN ( R5, M, MIDPOINT (E.b) )

BETWEEN ( R3, R1, R5 )

EQUAL ( DISTANCE (R1, R3), DISTANCE (R3, R5) )

EQUAL ( DISTANCE (R1, AXIS1), DISTANCE (R3, AXIS1) )

EQUAL ( DISTANCE (AXIS1, R3), DISTANCE (AXIS1, R5) )

EXCLUDE X ( CIRCLE )

INSIDE ( X, E )

PARAMETERS GRASP (POSITION)

GRASP.POINT = E.CENTROID

GRASP.ANGLE = E.a.ANGLE

#OBJ BLOCK22

CONTOURS E (BLOCK), M (RECTANGLE), L1, L2, R1, R2 (CIRCLE)

INSIDE ( M, E)

INSIDE ( L1, E)

INSIDE ( L2, E)

INSIDE ( R1, E)

INSIDE ( R2, E)

GREATER ( LENGTH (E.a), LENGTH (E.b) )

LESS ( LENGTH (M.a), LENGTH (M.b) )

PARALLEL ( M.a, E.a )

AUX AXIS1 = LINE ( MIDPOINT (E.d), MIDPOINT (E.b) )

AUX AXIS2 = LINE ( MIDPOINT (E.a), MIDPOINT (E.c) )

BELONGS ( MIDPOINT ( M.a), AXIS2 )

BETWEEN ( M, L1, R1 )

BETWEEN ( M, L2, R2 )

AUX L = LINE ( L1.a.CENTER, L2.a.CENTER )

AUX R = LINE ( R1.a.CENTER, R2.a.CENTER )

PARALLEL ( L, R )

AUX PL = INTERSECTION ( L, AXIS1 )

BETWEEN ( PL, L1, L2 )

EQUAL ( DISTANCE ( L1, PL ), DISTANCE (L2, PL ))

AUX PR = INTERSECTION ( R, AXIS1 )

BETWEEN ( PR, R1, R2 )

EQUAL ( DISTANCE ( R1, PR ), DISTANCE (R2, PR ))

EQUAL ( DISTANCE ( L1, PL ), DISTANCE (R1, PR ))

EXCLUDE X ( CIRCLE )

INSIDE ( X, E )

PARAMETERS GRASP (POSITION)

GRASP.POINT = E.CENTROID

GRASP.ANGLE = E.a.ANGLE

#OBJ BLOCK43

CONTOURS E (BLOCK), M (RECTANGLE), L1, L2, L3, L4, R1, R2, R3 (CIRCLE)

INSIDE ( M, E)

INSIDE ( L1, E)

INSIDE ( L2, E)

INSIDE ( L3, E)

INSIDE ( L4, E)

INSIDE ( R1, E)

INSIDE ( R2, E)

INSIDE ( R3, E)

GREATER ( LENGTH (E.a), LENGTH (E.b) )

LESS ( LENGTH (M.a), LENGTH (M.b) )

PARALLEL ( M.a, E.a )

AUX AXIS1 = LINE ( MIDPOINT (E.d), MIDPOINT (E.b) )

AUX AXIS2 = LINE ( MIDPOINT (E.a), MIDPOINT (E.c) )

BELONGS ( MIDPOINT ( M.a), AXIS2 )

SYMMETRICAL ( L1, L2, AXIS1 )

SYMMETRICAL ( L3, L4, AXIS1 )

AUX R = LINE ( R1.a.CENTER, R3.a.CENTER )

BELONGS ( R2.a.CENTER, R )

BELONGS ( R2.a.CENTER, AXIS1 )

BETWEEN ( R2.a.CENTER, R1.a.CENTER, R3.a.CENTER )

EQUAL ( DISTANCE (R1, R2), DISTANCE (R2, R3) )

BETWEEN (R1, M, MIDPOINT (E.b) )

BETWEEN (R3, M, MIDPOINT (E.b) )

BETWEEN (L1, M, MIDPOINT (E.d) )

BETWEEN (L2, M, MIDPOINT (E.d) )

BETWEEN (L3, M, MIDPOINT (E.d) )

BETWEEN (L4, M, MIDPOINT (E.d) )

EQUAL ( DISTANCE (L1, L2), DISTANCE (L3, L4) )

EQUAL ( DISTANCE (L1, L3), DISTANCE (L2, L4) )

PARAMETERS GRASP (POSITION)

GRASP.POINT = E.CENTROID

GRASP.ANGLE = E.a.ANGLE
